# Broadband Balanced-to-Balanced Filtering Power Divider Using HMSIW-SSPP Transmission Line

**DOI:** 10.3390/mi15030358

**Published:** 2024-02-29

**Authors:** Hao Liu, Bing Xue, Jun Xu

**Affiliations:** 1 School of Physics, University of Electronic Science and Technology of China, Chengdu 611731, China; uestcliuhao@163.com (H.L.); xujun@uestc.edu.cn (J.X.); 2 Department of Electronics and Nanoengineering, School of Electrical Engineering, Aalto University, 02150 Espoo, Finland

**Keywords:** balanced-to-balanced structure, filtering power divider, broadband, half-mode substrate-integrated waveguide, spoof surface plasmon polariton

## Abstract

In this paper, a novel broadband balanced-to-balanced (BTB) filtering power divider (FPD) utilizing the half-mode substrate-integrated waveguide and spoof surface plasmon polariton (HMSIW-SSPP) hybrid transmission line is introduced. Initially, a new HMSIW-SSPP unit cell is proposed, demonstrating a lower upper cut-off frequency compared to the classical HMSIW-SSPP unit cell. Building upon this unit cell, a bandpass BTB FPD is devised employing dual-layer stacked substrates, enabling independent control over the passband’s lower and upper cut-off frequencies through specific physical dimensions. Additionally, the incorporation of isolation resistors and defected ground structures in the BTB FPD enhances differential-mode isolation and common-mode (CM) suppression between output ports. A manufactured and tested BTB FPD prototype validates this design method, showcasing a broad fractional bandwidth of 52.31% (6.72–11.48 GHz), output port isolation surpassing 14.25 dB, and transmitted CM suppression exceeding 34.05 dB.

## 1. Introduction

With the rapid advancements in modern wireless electronic technology, highly integrated microwave circuits, which combine filters with other passive devices to achieve multiple functions, have become a prominent research field. Among these, the balanced-to-balanced (BTB) filtering power divider (FPD) not only combines the functionalities of a power divider (PD) and bandpass filter (BPF) but also incorporates balanced input and output to counteract environmental noise. This makes it a crucial passive device for RF/microwave circuits. Previous studies have proposed BTB FPDs based on microstrip resonators [1,2] and patch resonators [3,4], showing good performance in common-mode (CM) suppression and featuring a straightforward design. However, these designs suffer from limitations in power capacity and increased losses at higher frequencies. Recently, an ultra-wideband BTB FPD with a high CM suppression level has been proposed based on hybrid microstrip and slotline spoof surface plasmon polariton (SSPP) structure [5], since the SSPP features unique characteristics, including strong field confinement, controllable dispersion, slow-wave effect, and lowpass characteristics. However, the SSPP BTB FPD in [5] also exhibits limitations of power and loss at higher frequencies. Also, its in-band return loss (RL) and isolation should be further improved.

The substrate-integrated waveguide (SIW) boasts high power capacity and easy integration with planar circuits. Several BTB FPDs based on SIW/half-mode SIW (HMSIW) resonators are documented in [6,7,8,9]. Ref. [6] introduced a dual-layer BTB FPD with robust CM suppression and high selectivity, utilizing TE_102_ mode resonators and cross-coupling technology. Ref. [7] reported a single-layer BTB FPD employing hybrid TE_101_-mode and TE_102_-mode cavities. In [8], a TE_120_-mode BTB FPD using right triangle resonators is proposed. Additionally, Ref. [9] suggested a compact single-layer BTB FPD by integrating one full-mode SIW cavity resonating at TE_210_ mode and two HMSIW cavities resonating at TE_120_ modes. Single-layer and dual-layer dual-band SIW BTB FPD with high performance are proposed in [10,11]. Despite these advancements, the mentioned BTB FPDs exhibit narrow bandwidths, lacking independent control of lower and upper cut-off frequencies of the passband, thus limiting their application scope.

To address this challenge, researchers have leveraged the SIW/HMSIW and SSPP (SIW-/HMSIW-SSPP) transmission line, resulting in the development of broadband bandpass filtering passive components like BPFs [12,13,14,15,16,17], balanced BPF [18], FPDs [19,20], filtering balun [21], diplexer [22], and filtering magic-T [23], enabling independent adjustment of lower and upper cut-off frequencies in the passband. Despite these advancements, to date, no literature has reported BTB FPDs based on SIW-SSPP or HMSIW-SSPP. Hence, this paper introduces the application of the HMSIW-SSPP transmission line to create a novel broadband BTB FPD. Furthermore, the integration of isolation resistors and defected ground structures (DGSs) in the BTB FPD enhances differential-mode (DM) isolation and CM suppression between output ports.

The arrangement of this paper is as follows. The dispersion properties of the proposed HMSIW-SSPP unit cell are analyzed in Section 2. The design process of the proposed BTB FPD, including enhancing DM isolation and CM suppression, is given in Section 3. Section 4 details the experiments and discussion of the results. The conclusions are presented in Section 5.

## 2. Characteristics of the HMSIW-SSPP

**Figure 1:**
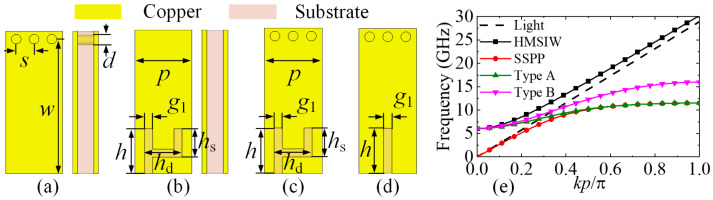
Unit cells’ configurations and simulated dispersion curves. (**a**) HMSIW. (**b**) SSPP. (**c**) Type A. (**d**) Type B. (**e**) Dispersion curves of the four unit cells. (*d* = 0.60, *g*_1_ = 0.50, *h* = 2.75, *h*_d_ = 2.30, *h*_s_ = 1.75, *p* = 3.50, *s* = 1.10, *w* = 8.30; unit: mm).

Figure 1a shows the classical HMSIW. The thickness of the substrate and metal is 0.508 mm and 18 µm, respectively. Figure 1b demonstrates the geometry of the proposed new SSPP unit cell based on a two-branch groove. The long transverse groove length is *h*, and the short one’s length is *h*_s_. The *h*_d_ represents the longitudinal groove length. The *g*_1_ represents the width of the groove. Figure 1c illustrates the geometry of the proposed HMSIW-SSPP unit cell (Type A). The HMSIW and SSPP structures are merged to create Type A. When *h*_d_ = *g*_1_, Type A degenerates into a classical HMSIW-SSPP unit cell (Type B), as demonstrated in Figure 1d. The period of these unit cells is denoted as *p*. Figure 1e gives the simulated fundamental-mode dispersion curves of HMSIW, SSPP, Type A, and Type B. The dispersion curve of light is also given as a reference. All the unit cells are analyzed based on Rogers5880 substrate with *ε*_r_ = 2.2. As depicted in Figure 1e, the HMSIW has a highpass performance, while the new SSPP structure exhibits a lowpass characteristic. Due to the combination of HMSIW and SSPP structure, Type A exhibits a bandpass characteristic with frequencies ranging from 6.02 to 11.52 GHz, showing a lower upper cut-off frequency than Type B as the periods and long lateral groove lengths are the same. By using this HMSIW-SSPP hybrid transmission line, we can easily design a bandpass filtering component with a compact structure.

**Figure 2:**
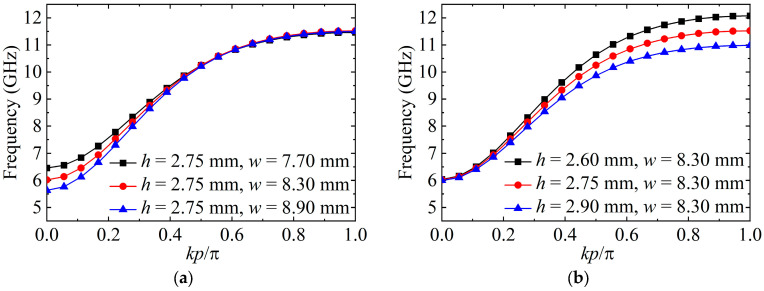
Dispersion curves of Type A under the effects of various (**a**) *w* and (**b**) *h*.

Figure 2a,b show the relationship between Type A’s dispersion curves and the structural parameters (*w* and *h*). With the rise in *w*, Type A’s lower cut-off frequency lowers. Instead, its upper cut-off frequency still serves as a constant. As *h* increases, Type A’s upper cut-off frequency decreases while the lower cut-off frequency is almost unaltered. Consequently, we can independently manipulate Type A’s upper and lower cut-off frequencies.

## 3. Design of HMSIW-SSPP BTB FPD

### 3.1. Design of HMSIW BTB PD

**Figure 3:**
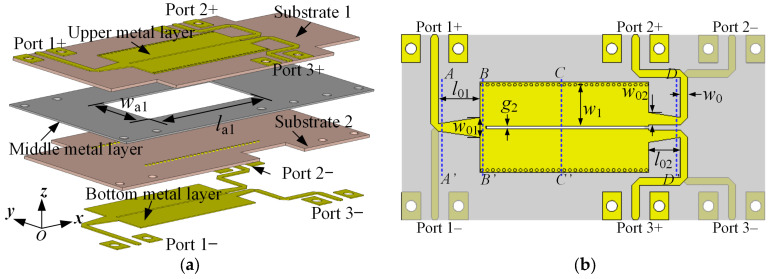
Schematic of the HMSIW BTB PD. (**a**) Explosion view. (**b**) Top view (Substrates 1 and 2 are not displayed). (*g*_2_ = 0.50, *w*_0_ = 1.50, *w*_1_ = 8.30, *l*_01_ = 8.30, *l*_02_ = 6.30, *l*_a1_ = 32.24, *w*_01_ = 4.00, *w*_02_ = 2.50, *w*_a1_ = 15.50; unit: mm).

Figure 3 shows the schematic representation of the dual-layer HMSIW BTB PD. It comprises two layers of substrate and three layers of metal. Two rows of periodically arranged metallic vias run through the entire multi-layer structure and connect the upper and bottom layers of metal. Port 1+ and 1− are balanced input ports, sharing the intermediate ground plane. The longitudinal long slot located in the bottom and upper layers of metal divides the SIW into two symmetrical HMSIW structures and achieves equal power distribution. Port 2+ and 2− (3+ and 3−) serve as the balanced output of Port 2 (3). Port 2+ (3+) shares the intermediate ground plane with Port 2− (3−). The DM signal transmission and CM signal suppression are mainly accomplished through the rectangular aperture (*w*_a1_ × *l*_a1_) in the middle metal layer [18].

**Figure 4:**
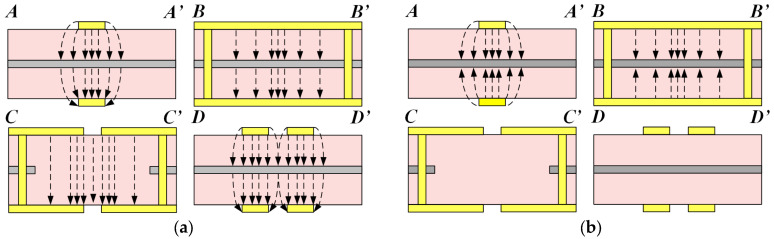
Sectional views of E-field distributions for the HMSIW BTB PD at different positions under (**a**) DM operation and (**b**) CM operation.

To further investigate the operating principle of HMSIW BTB PD, Figure 4a,b provide the schematic diagram of the electric-field (E-field) distributions under DM and CM excitations, respectively [18]. When Port 1+ and 1− are simultaneously excited with a 180° phase difference (DM driven), the DM signal’s E-field has the identical direction in the dual-layer structure, as shown in the A-A’ and B-B’ planes in Figure 4a. The longitudinal slot divides the DM signal into two channels transmitted in the HMSIW. The rectangular aperture etched in the middle metal layer acts as a virtual ground and has no impact on the transmission of the DM signal, as shown in C-C’ plane in Figure 4a. Then, employing the HMSIW-to-microstrip transition, a 180° phase difference is realized between Port 2+ (3+) and 2− (3−). Four microstrip ports provide two balanced output ports, as shown in D-D’ plane in Figure 4a. When Port 1+ and 1− are simultaneously excited at a 0° phase difference (CM-driven), the CM signal’s E-field in the dual-layer structure points toward the middle metal layer, as shown in the A-A’ and B-B’ planes in Figure 4b. The E-field in the dual-layer structure will be canceled when the CM signal passes through the middle metal layer’s aperture. Therefore, the CM signal is suppressed, as shown in the C-C’ and D-D’ planes in Figure 4b.

**Figure 5:**
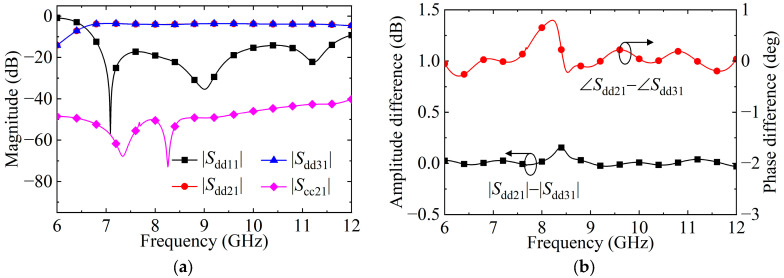
Simulated results of HMSIW BTB PD. (**a**) DM and CM S-parameters. (**b**) DM phase and amplitude differences.

The simulated DM and CM S-parameters of the HMSIW BTB PD are depicted in Figure 5a. It has a highpass transmission property under DM operation. In addition, the transmitted CM suppression is achieved in a broad frequency range. Figure 5b shows the simulated DM phase and amplitude differences between the balanced output ports, which are 0.8° and 0.16 dB, respectively. Thus, the equal-power and in-phase DM signals between the balanced Ports 2 and 3 are achieved. The HMSIW BTB PD’s simulated results are in line with the E-field distributions in Figure 4.

### 3.2. Design of HMSIW-SSPP BTB FPD I

**Figure 6:**
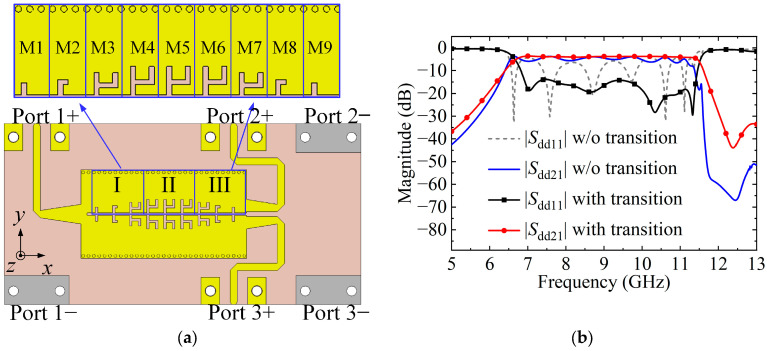
(**a**) Configuration of BTB FPD I. (**b**) Simulated |*S*_dd11_| and |*S*_dd21_| of the BTB FPD I with and without transition.

To realize the filtering function, we apply the proposed HMSIW-SSPP structure to the HMSIW BTB PD. Figure 6a demonstrates the structure of the HMSIW-SSPP BTB FPD (BTB FPD I). It is created by periodically inserting lowpass SSPP unit cells into the highpass HMSIW BTB PD to achieve the bandpass filtering function. The structure that performs the filtering function primarily consists of three sections: Part I, Part II, and Part III. Three HMSIW-SSPP unit cells (M1–M3) with progressively bigger grooves form Part I. Part II is the uniform HMSIW-SSPP structure composed of three identically sized HMSIW-SSPP unit cells (M4–M6). Three HMSIW-SSPP unit cells (M7–M9) with gradually decreasing groove sizes make up Part III. The values of the physical dimensions for M1 to M9 corresponding to Figure 1c are listed in Table 1. The function of Part I (III) is to achieve the transition from HMSIW (uniform HMSIW-SSPP) to uniform HMSIW-SSPP (HMSIW) structure. The simulated |*S*_dd11_| and |*S*_dd21_| of the BTB FPD I with transition and without transition (where the sizes of M1 to M9 are consistent with the M4) are given in Figure 6b. The BTB FPD I with transition attains an in-band |*S*_dd11_| enhancement over 9.4 dB compared to that without transition.

### 3.3. DM Isolation Enhancement between the Output Ports

**Figure 7:**
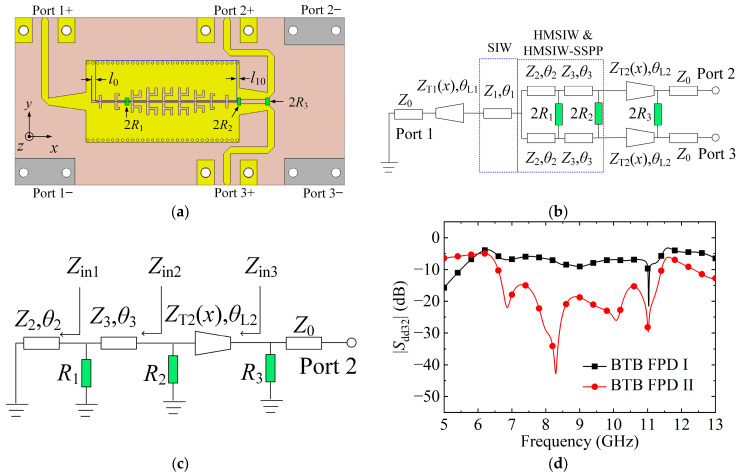
(**a**) Configuration of BTB FPD II. (**b**) Equivalent circuit of BTB FPD II’s half bisection under DM operation. (**c**) Odd-mode equivalent circuit of BTB FPD II’s half bisection under DM operation. (**d**) Simulated |*S*_dd32_| of the BTB FPDs I and II. (*l*_0_ = 0.82, *l*_10_ = 0.30; unit: mm).

To enhance the DM isolation of the balanced Ports 2 and 3, six resistors are attached to the BTB FPD I to obtain the BTB FPD II, as illustrated in Figure 7a. Its equivalent circuit of half bisection during DM operation is demonstrated in Figure 7b. Figure 7c is the odd-mode equivalent circuit of BTB FPD II’s half bisection under DM operation. The *θ*_2_ indicates the electrical lengths of the transmission structures between the SIW and resistor 2*R*_1_. This can be calculated by *θ*_2_ = *k*_0_*l*_0_ + *k*_1_*p*_1_ + *k*_2_*p*_2_ = 1.57 ≈ π/2, where *k*_0_, *k*_1_, and *k*_2_ are the propagation constants of the HMSIW, unit cells M1, and M2. Table 2 lists the values of *k*_0_, *k*_1_, and *k*_2_. The *l*_0_ is the length of the HMSIW at the left end of the structure. The *p*_1_ and *p*_2_ are the periods of the unit cells M1 and M2, as listed in Table 1. Then, the *Z*_in1_ approaches infinity. The *θ*_3_ indicates the electrical length of the transmission structures between resistors 2*R*_1_ and 2*R*_2_. The *Z*_in2_ can be written as:

(1) $$Z_{\mathrm{in}2}=Z_{3}\frac{R_{1}+jZ_{3}\tan\theta_{3}}{Z_{3}+jR_{1}\tan\theta_{3}}$$

Here, *θ*_3_ can be calculated by:

(2) $$\theta_{3}\;=\;k_{0}l_{10}+\sum_{3}^{n}k_{n}p_{n}=6.2823\approx 2\pi \;(n=3,4,\ldots 9),$$

where *p_n_* and *k_n_* are the periods and propagation constants of the unit cells Mn, as listed in Table 1 and Table 2, respectively. The *l*_10_ is the length of the HMSIW at the right end of the structure. Then, Equation (1) can be reduced to *Z*_in2_ = *R*_1_. The length of the output gradient microstrip line is *l*_02_ = 6.30 mm, which is approximately a quarter effective wavelength at 8.7 GHz. The gradient microstrip line’s characteristic impedance can be calculated using the following equations [24]:

(3) $$Z_{T2}(x)=\frac{120\pi }{\sqrt{\varepsilon_{e}}[w_{1}(x)/h+1.393+0.677\ln(w_{1}(x)/h+1.444)]}$$

and

(4) $$w_{1}(x)=\frac{x(w_{02}-w_{0})+w_{0}l_{02}}{l_{02}}$$

To simplify the calculation, we set *R*_2_ = *R*_1_ and select the *x* = 3.15 as the gradient microstrip line’s characteristic impedance. Then, the expression of *Z*_in3_ is:

(5) $$Z_{\mathrm{in}3}=Z_{T2}(x=3.15)\frac{(R_{1}/2)+jZ_{T2}(x=3.15)\tan\theta_{L2}}{Z_{T2}(x=3.15)+j(R_{1}/2)\tan\theta_{L2}}$$

Here, *θ*_L2_ ≈ π/2. Then, the *Z*_in3_ ≈ 4560/*R*_1_.

To meet Port 2′s good impedance matching, the following equation should be satisfied:

(6) $$Z_{0}=\frac{Z_{\mathrm{in}3}R_{3}}{Z_{\mathrm{in}3}+R_{3}}$$

Here, we selected *R*_1_ = 45.6 Ω and *R*_3_ = 100 Ω. In practical application, the final values of the isolation resistors are 2*R*_1_ = 91 Ω, 2*R*_2_ = 91 Ω, and 2*R*_3_ = 200 Ω. The simulated |*S*_dd32_| of the BTB FPDs I and II are illustrated in Figure 7d. The simulated in-band isolation between Ports 2 and 3 of BTB FPD II achieves around 8.8 dB enhancement compared to BTB FPD I.

### 3.4. Improvement in CM Suppression between the Output Ports

**Figure 8:**
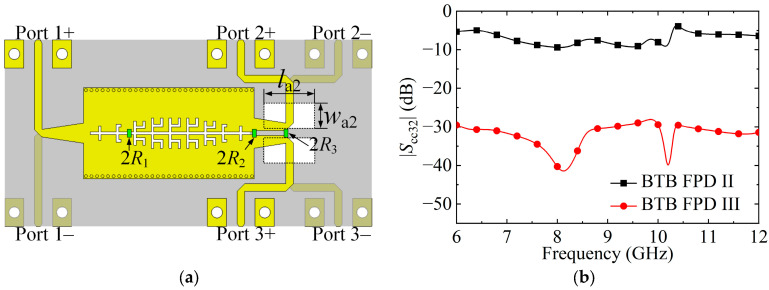
(**a**) Schematic of the BTB FPD III. (**b**) Simulated |*S*_cc32_| of BTB FPDs II and III. (*l*_a2_ = 10, *w*_a2_ = 5; unit: mm).

To further improve CM suppression between Ports 2 and 3, two DGSs (*w*_a2_ × *l*_a2_) are embedded in the middle metal layer of BTB FPD II to obtain BTB FPD III, as shown in Figure 8a. The simulated |*S*_cc32_| of BTB FPDs II and III are plotted in Figure 8b. As observed, the isolated CM suppression of BTB FPD III is better than that of BTB FPD II, because two DGSs can cancel out the CM signal’s E-field in dual-layer structure to block the transmission of the CM signal between Ports 2 and 3.

**Figure 9:**
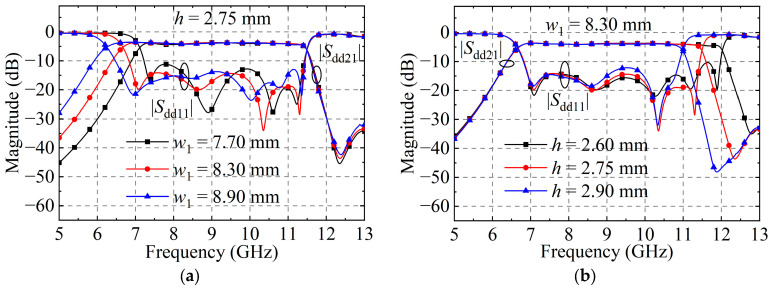
Simulated |*S*_dd11_| and |*S*_dd21_| of the BTB FPD III as a function of (**a**) *w*_1_ and (**b**) *h*.

Furthermore, the effects of BTB FPD III structural parameters on the |*S*_dd11_| and |*S*_dd21_| are studied, as illustrated in Figure 9. As the HMSIW’s width increases, the lower cut-off frequency of the passband shifts down, while the upper cut-off frequency is almost unaltered. As the long lateral groove of the unit cells M4–M6 lengthens, the passband’s upper cut-off frequency shifts downwards, and the lower cut-off frequency remains almost unaltered. Thus, the lower and upper cut-off frequencies of the passband can be flexibly and independently controlled.

The following is a summary of the proposed BTD FPD’s design procedures.

(1) Determine the sizes of the unit cell Type A. Based on the intended frequency response, the width *w* and the groove’s length *h* of Type A can be determined according to the independently adjustable characteristics of Type A in Figure 2.
(2) Design the HMSIW BTB PD. The HMSIW BTB PD is constructed based on the width *w* of Type A to obtain a highpass BTB frequency response, as plotted in Figure 5.
(3) Design the HMSIW-SSPP BTB FPD. The HMSIW-SSPP BTB FPD can be designed by loading three SSPP unit cells with the groove length of *h* and transition structures (Part I and Part III in Figure 6a) into HMSIW BTB PD. The initial groove lengths of unit cells in the transition structures change linearly from M1 to M4 and M6 to M9. After optimization by simulation software, good impedance matching can be achieved (Figure 6b).
(4) Improvement in DM isolation between output ports. The values and positions of the isolation resistors can be obtained based on Formulas (1)–(6) and propagation constants of each unit cell calculated by eigenmode analysis.
(5) Improvement in CM suppression between output ports. The initial length of output port DGS is set to three times the width of the microstrip line, and then it is further optimized to make |*S*_cc32_| approach −30 dB.

It is worth noting that the lower cut-off frequency of the final HMSIW-SSPP BTB FPD is slightly higher than that of unit cell Type A. This can be fine-tuned according to the independently adjustable characteristics in Figure 9 to meet the initial intended frequency response.

**Figure 10:**
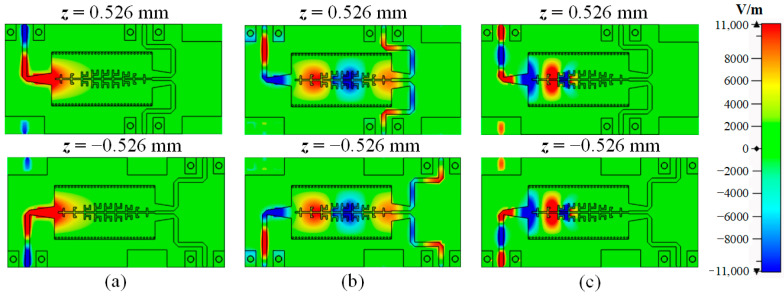
Simulated E-field distributions of proposed BTB FPD III at different frequencies. (**a**) 5.0 GHz. (**b**) 8.7 GHz. (**c**) 13.0 GHz. The coordinate origin is at the center of the rectangular aperture.

Figure 10 gives the simulated E-field distributions of BTB FPD III at various frequencies for more investigation of the transmission characteristics. At 8.7 GHz (the in-band frequency), the input DM signal can effectively transmit and convert into two equal DM output signals. At the lower stopband frequency of 5.0 GHz, the highpass characteristic of HMSIW blocks the input DM signal. When the frequency is at 13.0 GHz (the upper stopband frequency), the DM signal passes through Part I of the BTB FPD III, since it is still lower than the cut-off frequencies of HMSIW and unit cells M1–M3. However, the DM signal is reflected by Part II, since it is outside the cut-off frequency of unit cells M4–M6.

## 4. Experiments and Discussion

**Figure 11:**
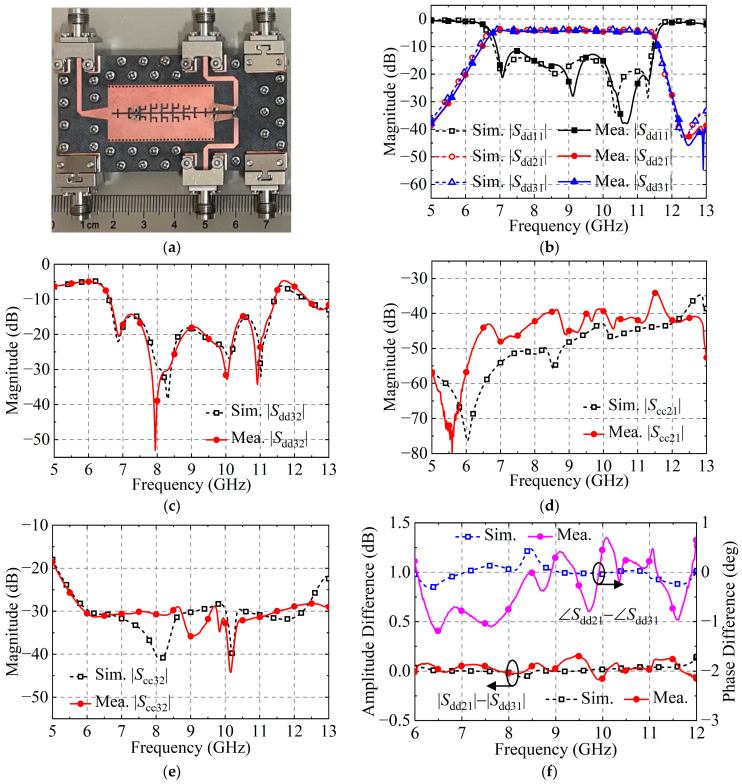
(**a**) Fabricated prototype of the proposed BTB FPD III. (**b**) Simulated and measured |*S*_dd11_|, |*S*_dd21_|, and |*S*_dd31_|. (**c**) Simulated and measured |*S*_dd32_|. (**d**) Simulated and measured |*S*_cc21_|. (**e**) Simulated and measured |*S*_cc32_|. (**f**) Simulated and measured DM amplitude and phase difference.

Figure 11a demonstrates the fabricated prototype of the BTB FPD III composed of dual-layer substrates, which are manufactured individually using the normal printed circuit board process and then fixed with multiple screws and nuts. The substrate is Rogers5880 with *ε*_r_ = 2.2, tan*δ* = 0.0009, and a thickness of 0.508 mm. The overall circuit size of the proposed BTB FPD is 73.0 mm × 37.1 mm (3.28 *λ*g × 1.67 *λ*g). Here, *λ*g = c/*f*_0_/*ε*_r_^0.5^. The cost of this manufacturing way is lower than that of the integrated process. In measurements, six end-launched adapters (DC–50 GHz) are attached to the fabricated prototype using screws for connection with the two-port vector network analyzer (VNA) R&S^®^ZNA50. The IF bandwidth of VNA is set to 1 KHz. The scanning frequency range is 5–13 GHz with scanning points 801. The single-end S-parameters are measured with the supports of four 50-Ω terminators (DC–40 GHz). Then, the mixed S-parameter method [25] is utilized to convert the measured single-end S-parameter into the required DM and CM S-parameters. Figure 11b,c show the measured and simulated results of the DM S-parameters. The measured 3 dB FBW is 52.31% (6.72–11.48 GHz). The measured in-band minimum insertion loss (IL) excluding the 3 dB loss from PD is 1.00 dB. The measured |*S*_dd11_| is <−11.57 dB in the passband. Moreover, in-band isolation between Ports 2 and 3 is higher than 14.25 dB. Figure 11d,e demonstrate simulated and measured results of the CM S-parameters. The measured |*S*_cc21_| is <−34.05 dB from 5.00 to 13.00 GHz. In addition, the measured |*S*_cc32_| is <−28.00 dB from 6.00 to 13.00 GHz. The simulated and measured DM phase and amplitude differences are plotted in Figure 11f. The measured in-band amplitude difference is 0.16 dB, and the measured in-band phase difference is 1.1°. There are good agreements between the simulation and measurement.

In practical application, the position of balanced feeding lines makes the proposed BTB FPD more suitable as a module equipped with coaxial cable adapters. A conversion structure can be designed to make the balanced feeding lines in the same metal layer if it is employed in an integrated application scenario. In addition, using screws and nuts to assemble PCBs may cause additional assembly errors, and it is time-consuming to optimize the transition structures for good impedance matching. This design method can be extended to implementing a dual-band HMSIW-SSPP BTB FPD.

Table 3 shows the performance of the proposed BTB FPD and other reported BTB FPDs. The proposed BTB FPD based on the HMSIW-SSPP transmission line has a broader operating bandwidth than the BTB FPDs in [2,3,4,6,7,8,9], and it also possesses independent control of the passband’s lower and upper cut-off frequencies, making obtaining the desired frequency response easier. Although the SSPP BTB FPD in [5] achieves a 152% FBW and compactness, this proposed design has better IL, RL, and isolation than that of [5]. The proposed BTB FPD’s DM isolation between output ports approaches the BTB FPDs in [8,9], but within a broader range. This design features a better IL than that of BTB FPDs in [2,3,4,5,6,7,8,9].

## 5. Conclusions

This paper introduces a novel broadband BTB FPD utilizing an HMSIW-SSPP transmission line. A comprehensive overview of the HMSIW-SSPP BTB FPD design and test has been presented. The simulations and measurements of the BTB FPD are in good agreement, showcasing a broad fractional bandwidth of 52.31% (6.72–11.48 GHz), output port isolation surpassing 14.25 dB, and transmitted CM suppression exceeding 34.05 dB. The proposed BTB FPD exhibits the advantages of high CM suppression, low loss, and high DM isolation. Compared to previously reported BTB FPDs using SIW/HMSIW resonators, this design offers a broader bandwidth and simpler bandwidth control. Its potential as a valuable application for future broadband balanced wireless communication systems is noteworthy.

## Figures and Tables

**Table 1:** Physical dimensions of M1 to M9 (unit: mm).

Unit Cell	*h*	*h*_d_	*h*_s_	*g*_1_	*p*
M1 (M9)	1.20	-	-	0.50	3.50
M2 (M8)	1.50	1.00	-	0.50	3.50
M3 (M7)	2.14	2.30	1.45	0.50	3.50
M4–M6	2.75	2.30	1.75	0.50	3.50

**Table 2:** Propagation constants of HMSIW, unit cells M1 to M9 at 8.7 GHz (unit: rad/mm).

*k*_0_	*k*_1_, *k*_9_	*k*_2_, *k*_8_	*k*_3_, *k*_7_	*k*_4_, *k*_5,_*k*_6_
0.186	0.197	0.208	0.246	0.294

**Table 3:** Comparisons between this research and earlier reported BTB FPDs.

Reference	*f*_0_ (GHz)	3 dB FBW (%)	IL (dB)	RL (dB)	CMS (dB)	ISO (dB)	Difference (dB/°)	ICOCF	Size (*λ*_g_ × *λ*_g_)
Microstrip Resonators [2]	2.39	7	2.21	-	60	20	-/-	No	0.46 × 0.40
Patch Resonators [3]-1	1.8	18.2	1.08	-	52	29.5	0.05/0.2	No	0.55 × 0.55
Patch Resonators [4]	2.63	15.7	1.50	17.5	46	-	-/-	No	0.59 × 0.59
SSPP [5]	4.0	152	1.6	5.2	35	6 ^[S]^	-/-	Yes	1.45 × 1.27
SIW Resonators [6]	10	3.5	1.9	15	35	-	-/0.6	No	3.35 × 3.35
SIW Resonators [7]	10.6	4.62	1.01	16	40	-	0.3/4	No	3.82 × 2.12
SIW Resonators [8]	14.91	4.1	1.3	17	42	16.1	0.2/2	No	0.59 × 0.71
SIW + HMSIW Resonators [9]	10.5	6.8	1.2	14	26	15.0	-/1.5	No	-
HMSIW-SSPP TL (This)	9.1	52.31	1	11.57	34.05	14.25	0.16/1.1	Yes	3.28 × 1.67

TL: transmission line; CMS: common-mode suppression (|*S*_cc21_|); ^[S]^: simulated result; ISO: differential-mode isolation between Ports 2 and 3; Difference: Amplitude and phase difference between the two output ports; ICOCF: Independent controllability of cut-off frequencies.

## Data Availability

Data are contained within the article.
